# Dual-Layer Index for Efficient Traceability Query of Food Supply Chain Based on Blockchain

**DOI:** 10.3390/foods12112267

**Published:** 2023-06-05

**Authors:** Chaopeng Guo, Yiming Liu, Meiyu Na, Jie Song

**Affiliations:** Software College, Northeastern University, Shenyang 110000, China; guochaopeng@swc.neu.edu.cn (C.G.);

**Keywords:** traceability system, food supply chain, traceability query, blockchain, indexing

## Abstract

Blockchain techniques have been introduced to achieve decentralized and transparent traceability systems, which are critical components of food supply chains. Academia and industry have tried to enhance the efficiency of blockchain-based food supply chain traceability queries. However, the cost of traceability queries remains high. In this paper, we propose a dual-layer index structure for optimizing traceability queries in blockchain, which consists of an external and an internal index. The dual-layer index structure accelerates the external block jump and internal transaction search while preserving the original characteristics of the blockchain. We establish an experimental environment by modeling the blockchain storage module for extensive simulation experiments. The results show that although the dual-layer index structure introduces a little extra storage and construction time, it significantly improves the efficiency of traceability queries. Specifically, the dual-layer index improves the traceability query rate by seven to eight times compared with that of the original blockchain.

## 1. Introduction

Ensuring food safety is crucial to maintaining people’s quality of life. The food supply chain consists of the entire process of food production: processing, distribution, consumption, and disposal [1,2]. However, the lack of transparency and traceability can lead to large-scale food fraud affecting millions of people [3]. Transparency can enhance consumers’ confidence in food quality and safety, and traceability can be used to verify compliance with the food circulation process and prevent contamination or fraud in the supply chain [4]. Therefore, it is essential to ensure the transparency and traceability of the historical data in the food supply chain.

Traceability enables stakeholders to track products from the farm to the fork, ensuring safety and high quality [5]. Traceability queries for historical data can trace products in the supply chain and track the circulation of products [6]. For example, in the food supply chain, consumers can use traceability queries to obtain the source of food-related issues and any subsequent health problems they may have experienced. By providing a clear view of the entire supply chain, traceability helps to quickly identify issues, isolate contaminated products, and prevent the further spread of contamination or fraud.

Traditional centralized traceability systems have several issues, such as data monopoly, low security, untrustworthiness, low efficiency of data sharing, lack of transparency, and difficulty in meeting the needs of modern society [7]. Blockchain provides a new solution for traceability systems. The distributed storage structure of blockchain ensures the authenticity and integrity of information. Moreover, the decentralized platform and consensus mechanism prevent malicious tampering with information collection and circulation [8].

Blockchain can be broadly defined as a distributed database that shares and maintains data consistency. It consists of a connected sequence of blocks that store data in a block-and-chain structure. Transactions with timestamps are protected by public key encryption and verified by the community. There are four key features of blockchain. Firstly, it is decentralized. Transactions can be conducted between any two clients in the network without a central server or a third-party agent. Secondly, blockchain is persistent. Transactions cannot be altered after being verified and recorded [9]. Thirdly, blockchain is anonymous. Users can generate one or multiple anonymous addresses to conduct transactions, effectively avoiding personal identity exposure. Finally, blockchain is auditable. With every transaction being authenticated and recorded with a timestamp, users can track and verify records from any node in the network, enhancing the traceability and transparency of the stored data [10]. For a more detailed introduction to blockchain technology and its applications in the supply chain, please refer to the review article by Sunny et al. [11].

Blockchain provides traceability capabilities [12]. In the storage structure of a blockchain, the block header data contain the hash value of the previous block, which can be used to retrieve the previous block and enable retrospective query functionality through traversal. However, blockchain is not optimized for traceability or conditional queries, resulting in extremely low query efficiency [13]. To query records, blockchain has to traverse all records to generate the final query results [14]. Many blockchain systems, such as Fabric, Ethereum, and Parity, distinguish the current and historical state of data. Data related to various events are written in the form of key–value pairs. For key *k*, the data include the latest pair of the current state of *k* and all pairs of the historical state of *k*. The collection of current states for all keys is denoted as state-db, while the collection of the historical states is denoted as history-db. The history-db data are time-dependent in nature, and analysis of these data can generate valuable business insights and support various use cases [10]. In this study, we treated the query method of obtaining these historical data as traceability queries. Notably, traceability queries in the native blockchain must be conducted by traversing blocks, which is time-consuming.

Current research has mainly focused on blockchain conditional query optimizations, involving two research directions. The first is enriching the query function and improving the query efficiency with external databases. However, these approaches lose blockchain security and require additional storage management and cost. The second one is altering the block structure to achieve lower storage space overhead and keep the security mechanism. Generally, a blockchain traceability query includes two phrases: an internal block query and an external block jump. The internal block data query searches the data according to given query conditions, for example, the produce ID within a block. The external block jump searches the previous block that contains the corresponding historical records. Improvements in the conditional query can optimize the internal block query performance but not the external block jump. Therefore, the current blockchain conditional query optimization methods comprehensively cannot boost traceability queries.

In this study, we followed the structure alternation direction to optimize the traceability query performance by introducing a dual-layer index structure. The dual-layer index structure includes an internal index and an external index. The internal index reconstructs the storage structure of the block by combining a Merkle tree and a B+ tree to accelerate the internal block data query. The external index alters the storage structure of the transaction. It contains a temporary table of traceability objects to realize a direct jump between blocks according to the traceability target to optimize the external block jump. Through the combination of the internal index and the external index, the dual-layer index structure not only optimizes the conditional query of blockchain but also enhances traceability queries without losing the characteristics of blockchain.

The main contributions of this study are as follows:

(1) We addressed the issue of low traceability query efficiency in blockchain-based traceability systems and avoided the tamper resistance loss caused by introducing external databases. Our solution ensures both efficient querying and the integrity of the blockchain.

(2) The dual-layer index accelerates the two steps of traceability data retrieval, namely internal block search and external block jump. It comprehensively and effectively improves traceability query efficiency.

(3) The experimental results demonstrate that the dual-layer index structure improves traceability query efficiency by approximately seven to eight times compared with that of the native Merkle tree, making it suitable for blockchain-based traceability systems.

(4) We abstracted the storage layer of blockchains to build a blockchain storage module, providing a tool for stimulation experiments, in which we identified the jump cost between blocks in the traceability process as a factor affecting traceability efficiency, providing ideas for the subsequent optimization of traceability efficiency.

The organization of the paper is as follows: Section 2 summarizes related studies, including blockchain-based food supply chain studies, general supply chain studies, and blockchain-related query efficiency optimization approaches. Section 3 presents the methodology in our research. Section 4 describes the design of the dual-layer index structure in detail, and Section 5 illustrates the operations of the dual-layer index. Section 6 shows the experimental setup and results. Section 7 presents the discussion of our findings, including experimental result analysis, a comparison with related methods, implementation details, and a summary of the assumptions and limitations of our method. Section 8 concludes the paper.

## 2. Related Studies

The traditional centralized traceability system in the food supply chain lacks transparency and traceability. In contrast, blockchain technology has the potential to meet the transparency and traceability requirements of the food supply chain. However, the traceability query efficiency in blockchain can be a limiting factor for the overall efficiency of the food supply chain. This section introduces existing supply chain optimization methods based on blockchain and demonstrates the feasibility of using blockchain technology in the food supply chain. Furthermore, this section discusses optimization methods that rely on external databases and structural changes and compares their advantages and differences with those of the approach proposed in this paper.

### 2.1. Food Supply Chain Based on Blockchain

Prashar et al. [15] proposed a blockchain-based solution that eliminates the need for a secure centralized structure, resulting in a transparent, accurate, and traceable supply chain system. Damoska Sekuloska et al. [16] developed a three-layer model for a smart local food supply chain that enhances food accessibility, traceability, and safety. Zhang et al. [17] developed a trusted traceability system for grain and oil food using the Hyperledger Fabric framework. The system addresses issues related to low data security and poor data sharing while also improving traceability accuracy. Kechagias et al. [4] demonstrated an application that improves product traceability for producers by leveraging Ethereum to provide a secure, transparent, and efficient solution for tracking and tracing products in the supply chain.

The above studies have confirmed that blockchain technology provides a feasible technical solution for the food supply chain, which can improve the traceability, transparency, and safety of the food supply chain. However, the goals of the above studies slightly differ from ours. In this study, we tried to optimize the performance of traceability queries in the blockchain-based food supply chain while keeping the blockchain features.

### 2.2. Other Supply Chain Based on Blockchain

Kong et al. [18] proposed BCSChain based on blockchain for the ceramic supply chain system. BCSChain aims to address the issues of transparency and security prevalent in traditional ceramic supply chains. Hader et al. [19] introduced a novel traceability framework for the textile supply chain based on blockchain technology. This framework allows a transparent information-sharing platform among all supply chain members. Abdallah et al. [20] proposed a blockchain-based framework for the online sale of pharmacutical products. The system keeps track and informed about sale transactions and ensures secure payment dispersal and medicine delivery. Liu et al. [21] developed a financial management platform that merges blockchain and supply chain technologies. By utilizing blockchain credit transfer, information sharing, data traceability, and tamper-proof features, this platform aims to solve critical issues such as the inability to transfer core enterprise credit.

Several researchers have proposed new solutions that leverage the unique characteristics of blockchain. The results of these studies have demonstrated that blockchain can effectively address the security and traceability issues inherent in traditional supply chain systems.

### 2.3. Optimization Methods Based on External Database

One direction is to integrate database techniques into blockchain. Helmer et al. [22] proposed EternityDB, integrating database functions into blockchains via intelligent contracts, in which databases and data are moved to the blockchain. However, copying and storing such large databases on each node of a peer-to-peer network not only wastes storage space but also increases storage pressure and creates cost problems. BigChainDB [23] combines document-based NoSQL database functions to achieve the fast query and reliability of blockchain but lacks SQL support. Muzammal et al. [24] proposed ChainSQL, which supports SQL operations. However, like BigChainDB, their actual data reside in an off-chain database. Namely, users must trust their connected peers and ensure the database stores accurate data because the query results might not be consistent with the blockchain.

Another direction is to embed middleware in the blockchain to support database operations. Zhou et al. [25] proposed Ledgerdata to improve the query efficiency of super ledger blocks and transactions by adding data analysis middleware to Hyperledger Fabric. However, its application scope is limited to Fabric. Li et al. [26] proposed EtherQL, a query layer on the blockchain, which provides an efficient query primitive to expand query function and improve query efficiency. Fa et al. [27] further expanded and enhanced the query expansion function. However, this approach requires that the server always returns correct results, which is too restrictive in real-world scenarios. Peng et al. [14] proposed a Verifiable Query Layer (VQL) that adopts a three-tier architecture, including the underlying blockchain system, middleware layer, and application layer. The middleware provides efficient and verifiable query services to ensure the authenticity of the query results of the blockchain. However, the MongoDB introduced by the middleware layer still needs additional management, leading to storage overhead.

Optimizing the query efficiency of blockchains using external databases can lead to the loss of the security characteristics of the blockchain. Additionally, it introduces storage management costs and reduces transparency in the food supply chain, which are unacceptable. As a result, some researchers have proposed optimization strategies based on structural alteration to address these issues. Namely, these strategies improve query efficiency while maintaining the security and transparency characteristics of the blockchain.

### 2.4. Optimization Methods Based on Structure Alternation and Novel Data Forms

Introducing external query structures to alter the blockchain structure is an essential method for improving the performance of blockchain queries. Ma et al. [28] designed a blockchain-based log system with lightweight, compatible modules for existing blockchain platforms. This system employs a hierarchical timestamp structure that enables efficient range queries on timestamp fields. Ren et al. [29] proposed a Bitcoin-based model for Internet of Things data queries that uses the dual-combination Bloom filter (DCOMB) query method. This method transforms the hash computing capacity used for Bitcoin mining into query computing power, enhancing transaction query proficiency.

Another direction for optimizing the efficiency of blockchain is to improve the storage structure of blocks. Pei et al. [30] introduced an indexing technique based on Merkle Semantic Trie. They extracted semantic information from the data in the chain to construct an indexed structure on the consensus chain, allowing for the real-time and effective query of data on and off the chain. Niu et al. [31] improved the efficiency of transaction search by reorganizing the blockchain and constructing an index structure using prefix tree and ODS technology. This scheme enables efficient, privacy-preserving transaction queries based on the transaction hash and address, even for lightweight clients. He et al. [32] proposed the T-Merkle tree, a novel blockchain storage structure that improves the design of the Merkle tree, reduces storage costs, and enhances query efficiency. Xu et al. [33] proposed the MPT-Chain tree, combining the characteristics of the Patricia tree and Merkle tree. MPT-Chain guarantees distributed index consistency and query result accuracy.

The inherent characteristics of blockchain, including transparency and traceability, are crucial to food supply chains. Our research idea is based on optimizing the traceability query of historical data by modifying the storage structure of the blockchain while retaining its fundamental characteristics. To achieve this, we reconstructed the storage structure of block data and added both internal and external indices.

In contrast to previous methods [30,31,32,33], our approach not only enhances query efficiency by adding indices within each block but also complements this with external indices. We further leveraged index fields in a temporary traceability table and leaf nodes within blocks to facilitate jumping to the destination block and avoid sequential queries, further improving the traceability query speed.

The other optimization direction is to utilize novel data forms. New and emerging data formats can potentially improve the analysis process [34]. The storage format of products in the supply chain can be simplified as spatiotemporal data, where temporal data record the time of product changes, and spatial data record the flow direction of products. With spatiotemporal data, the system can support context queries, and anomalies in supply can be easily found. Differently, in this study, we mainly focused on traceability performance rather than context queries.

In summary, our approach provides an efficient solution for blockchain-based traceability systems that ensures both querying efficiency and the integrity of the blockchain.

## 3. Methodology

In this section, we describe our research methodology, including our motivation, chosen approach, idea overview, and experimental design.

### 3.1. Motivation

Blockchain technology is known for certain key characteristics, including distribution, decentralization, security, nontampering ability, and traceability, that can effectively address the trust issues experienced with traditional traceability systems. However, these benefits can be limited by the inefficient querying capability of the blockchain system. Therefore, our research objective was to develop an optimization approach to enhance the efficiency of blockchain traceability queries.

### 3.2. Chosen Approach

Previous research can be categorized into two directions: incorporating an external database for efficient querying through database technology and introducing an index structure to enhance efficiency by modifying the block structure. However, using an external database raises security concerns and undermines the security features of blockchain. This violates the security requirements of supply chain traceability systems. Instead, we propose introducing an index structure and modifying the blockchain structure to enhance query efficiency while maintaining the fundamental characteristics of blockchain.

### 3.3. Idea Overview

The data traceability process in blockchain involves two steps: traversing all blocks to find the target block and then searching all data within that block. To optimize these steps, we propose a dual-layer index structure that limits the number of traversed blocks and the amount of data traversed within blocks. The dual-layer index structure utilizes an external index to facilitate external block jumps and an internal index to enable internal block query.

### 3.4. Experimental Design

We used a quantitative analysis method to evaluate the effectiveness of our proposed dual-layer index structure in improving query efficiency through experimental validation. Because using index structures may introduce time and space overhead, we designed comparison experiments to evaluate the performance of the dual-layer index structure against that of the native block structure in terms of time overhead, space overhead, and query efficiency. To account for variations in data amount and block size, we created an experimental dataset with varying values of these parameters. Similarly, we create another experimental dataset that varies the block depth to investigate its impact on query efficiency. In addition, we constructed a large-scale dataset and multiple complex scenarios to validate the performance of the dual-layer index under more complicated condition scenarios. Based on the experimental results, we analyzed the impact of the data amount and block size on the time and space overhead of the dual-layer index structure as well as the influence of data amount, block size, and block depth on query efficiency. We evaluated the performance improvement of the traceability query using the dual-layer index structure from these results and identified its applicable scenarios and limitations.

## 4. Dual-Layer Index Structure

The block storage structure of blockchains is designed to ensure data accuracy through Merkle trees or their variants [35], because any modification to the block structure alters the header hash value, causing a mismatch with the next block. Moreover, transaction existence is verified by expanding the “light nodes” in a public blockchain environment [36]. Typically, the Merkle tree is integrated into the transaction data of a block, which consists of two main parts: block header and block body. The block header consists of the hash value of the previous block (preHash), the Merkle tree root, and the hash value of the current block (curHash). The block body contains the Merkle branch segment. Traceability queries can be performed by recursively searching for the previous block, starting from the last block that contains the newest traceability transaction using preHash. However, given the high search cost associated with scanning all blocks, this approach severely limits the performance of the traceability query system.

To support fast traceability queries, a dual-layer index structure is proposed. The dual-layer index structure consists of an external index and an internal index. The external index allows for direct jumps between traceability-related blocks, while the internal index enables fast transaction data queries inside a block. Utilizing both the external and internal index, the dual-layer index structure can significantly improve the query efficiency of blockchain-based traceability systems.

### 4.1. External Index Design

Using the food supply chain as an example, each product is assigned a unique identifier, such as a string of bar codes, QR codes, or other forms stored within the blockchain. Additionally, each transaction within the blockchain is assigned a unique TransactionID, which can be used to differentiate between transactions. In a native blockchain, traceability query information is accessed by comparing the TransactionID and cross-referencing product identifiers to identify the desired information. In the dual-layer index structure, a TraceID is used to query the blockchain. The TraceID is the storage form of the product identifier mentioned earlier. The term “TraceID” is used interchangeably with “product identifier” to improve clarity.

He et al. [32] proposed a T-Merkle tree and showed that intermediate nodes could store the necessary data by modifying the node storage structure. Based on this idea, we achieve part of the external index in the block body. The external index consists of two parts: an item inside the blocks of the blockchain and a temporary traceability object table. The leaf nodes of a block with the external index contain more comprehensive data than the native structure, including TransactionID, TraceID, Time, preTraceHash, Transaction, and Hash. preTraceHash refers to the hash value of that specific block, where the previous transaction data with the same TraceID are stored. By means of preTraceHash, we can directly jump to the previous block location containing the transactions with the same TraceID. A temporary traceability object table is introduced to find the newest traceability block when we update a block or conduct a traceability query. By changing the storage structure in the blockchain, sequential retrieval in the traceability query becomes a direct jump to the traceability-related blocks.

**Figure 1:**
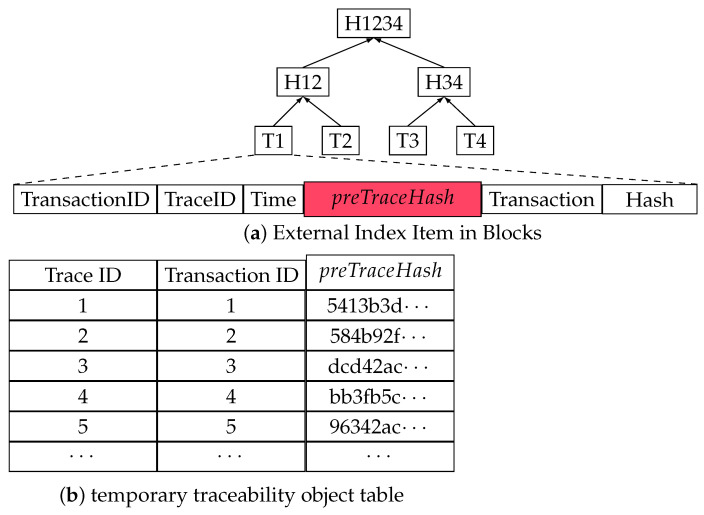
External index overview.

The external index overview is shown in Figure 1. Figure 1a shows the Merkle tree with the external index item. The leaf node (T1) stores TransactionID, TraceID, Time, preTraceHash, Transaction, and Hash. Both the intermediate node (H12) and root node (H1234) compute and store their own hash from the hash of their child nodes. Figure 1b shows a temporary table that stores the TraceID, TransactionID, and preTraceHash. By maintaining this temporary traceability object table, the initial traceability block can be quickly located.

Compared with the native Merkle tree, a Merkle tree with the external index structure shown in Figure 1 directly stores hash values at the leaf nodes, resulting in a shallower tree. This approach can accelerate the process of block jump during a traceability query by including the block hash value preTraceHash in the leaf node and maintaining the temporary traceability object table of traceability objects. However, implementing this external index structure for different blockchain types (e.g., Bitcoin, Ethereum, and Hyperledger Fabric) requires additional steps, such as adding temporary traceability tables and fields to transaction data.

In summary, the implementation of the external block index involves two parts: altering the storage structure of block internal transaction data and adding preTraceHash in the leaf node to enable a direct jump, and creating a temporary table to facilitate the fast retrieval of the newest traceability blocks to enhance the traceability query speed.

### 4.2. Internal Index Design

The B+ tree was shown to improve the speed of sequential lookup and support fast queries while reducing disk I/O [37]. Given the advantages of both the B+ tree and Merkle tree, we designed an MB+ tree (Merkle and B+ Tree) to support efficient traceability queries in the block. The MB+ tree leverages the tamper-proof features of Merkle trees to enable fast verification of blocks while also taking advantage of the efficient query and location capabilities of B+ trees to perform efficient searches of transaction data within blocks.

**Figure 2:**
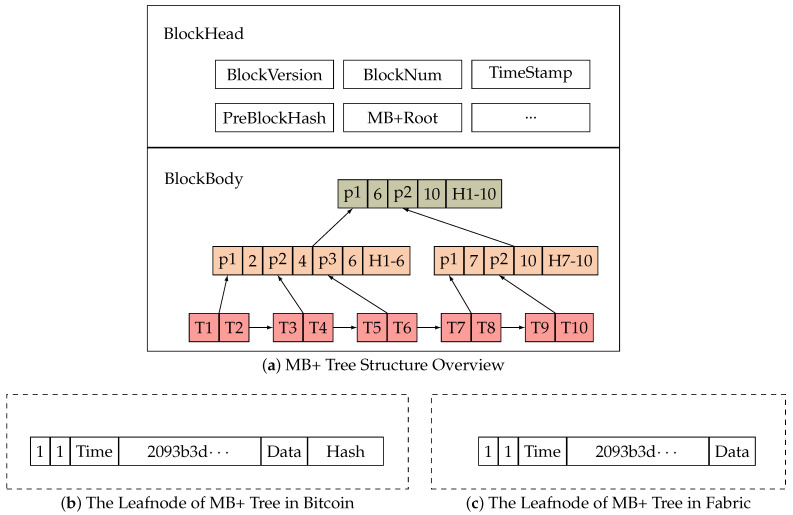
Block structure with internal index.

As shown in Figure 2a, the MB+ tree has all the characteristics of the B+ tree and the Merkle tree.

(1) In the B+ tree, keywords connect non-leaf nodes to their corresponding child nodes. Similarly, in the MB+ tree, TraceID is used as the keyword. (2) In the Merkle tree, leaf nodes store data, while other nodes store the hash of their child nodes. However, in the MB+ tree, the leaf node stores additional information, such as the TraceID, namely, the keyword required by the B+ tree, the preTraceHash required by the external index, and the hash value calculated by all the leaf node fields. The nonleaf nodes in the MB+ tree store pointers to their child nodes, keyword ranges, and hash values calculated by hashing all child nodes.

Specifically, in the MB+ tree, there are two types of nodes: leaf nodes and intermediate nodes (including the root node). Leaf nodes in the MB+ tree are responsible for storing all data. An example of the MB+ leaf node in Bitcoin is shown in Figure 2b, which consists of TransactionID, TraceID, Time, preTraceHash, Transaction, and Hash. The intermediate and root nodes in the MB+ tree (Figure 2a) are responsible for storing keyword information (2, 4, 6, 7, and 10), pointers (p1, p2, and p3), and hash values (H1–6) composed of keyword information, pointers, and child nodes.

In summary, based on the Merkle tree, we used the characteristics of the B+ tree to construct the MB+ tree to build the internal index. The keyword information and pointers are stored in the root and intermediate nodes. The leaf nodes store the block hash value (preTraceHash) of the previous traceability information and the fields of the TraceID as external index structures. By utilizing the pointers and keyword information, the traceability transaction data can be quickly located within a block.

Due to the different internal storage structures of specific blockchain architectures, such as Bitcoin, Ethereum, and Hyperledger Fabric, the internal indices of the three blockchains are discussed below.

In Bitcoin, the MB+ tree is utilized as shown in Figure 2a. The native Bitcoin leaf node stores transaction data, while the intermediate nodes, including the root node, store hash values. The MB+ tree leaf node, shown in Figure 2b, contains the TransactionID, TraceID, Time, Transaction, preTraceHash, and Hash. Additionally, the intermediate node stores a pointer that can point to the subnode and the keyword information representing the storage range of the su-node. Moreover, the leaf nodes are sequentially arranged and connected with a chain pointer. This design enables sequential data retrieval in the block based on the order of the TraceID.

The difference between using the MB+ tree in Ethereum and Bitcoin lies only in some of the data categories at the head of the block. However, the final MB+ tree data structure is the same as that for Bitcoin.

In Hyperledger Fabric, the FB+ tree is utilized as shown in Figure 2c. Similar to Bitcoin, the leaf node stores transaction data with additional details of signatures and transaction proposals. However, in contrast with Bitcoin, it no longer stores hash values. Figure 2c demonstrates the transaction content with additional details regarding the signature and transaction proposal. The intermediate and root nodes are responsible only for storing keyword information and pointers, with the keyword information represented by TraceID.

In summary, the implementation of the internal block index is primarily based on the features of the B+ tree, which was enhanced to form the MB+ tree. In the MB+ tree, the root and intermediate nodes store keyword information and pointers. The leaf nodes store the block hash value of the block where the previous traceability object is located along with an external index structure containing TraceID. Using pointers, the MB+ tree enables rapid retrieval of traceability transactions from a block.

## 5. Dual-Layer Index Operation

To take advantage of the dual-layer index structure, we altered the storage structure in Bitcoin, Ethereum, and Hyperlegder Fabric. Both Bitcoin and Ethereum use MB+ trees to store transaction data. Meanwhile, the FB+ tree is used in Hyperledger Fabric. The critical difference between the FB+ and MB+ trees is that the former no longer stores the hash value and omits the process of calculating the hash value, while most other structures remain unchanged. All three improved blockchains share the same external index structure. Therefore, creating and querying these indices are similar. In this section, we only consider Bitcoin as an example to illustrate the operations of the dual-layer index.

### 5.1. Dual-Layer Index Creation

During index creation, if the amount of data does not reach a predefined threshold, the data are initially stored in a cache table. The threshold is the maximum amount of transaction data a block can contain. Once we have enough initial data, a new block is created and linked to the previous block in the blockchain. The creation of the dual-layer index structure involves the creation of internal and external indices. In the following sections, we describe these two creation processes in detail.

#### 5.1.1. Internal Index Creation

The creation of the internal index involved creating an MB+ tree, in which nodes are arranged in an order set *M*. Building an MB+ tree includes three steps:

(1) When the first transaction is inserted into a new block, the system queries the temporary traceability object table associated with the external index structure of the block to obtain preTraceHash. Next, the system embeds the transaction data and preTraceHash into a leaf node, which becomes a root node later in the new block. During this process, the leaf node hash value LeafNodeHash is calculated based on the TransactionID, TraceID, Time, preTraceHash, and Transaction.

(2) When the other transaction data are inserted, the system queries the temporary traceability object table associated with the external index structure to obtain preTraceHash. The system embeds the transaction data into a leaf node and determines their insertion position based on the TraceID. If the number of keywords in the current node is less than or equal to |M|−1, the insertion is completed. Otherwise, the leaf node must be split into left and right leaf nodes. The left leaf node contains the data stored in the first |M|/2 leaf nodes, and the right leaf node stores the remaining data. The middle keyword, located at position |M|/2+1, is assigned to the parent node’s keyword. The parent node’s left pointer points to the left leaf node, and its right pointer points to the right leaf node.

(3) For the intermediate node, if the number of keywords is less than or equal to |M|−1, then the insertion is complete. Otherwise, the system must split the middle node into two intermediate nodes. The left intermediate node contains the first |M−1|/2 keywords, and the right node contains the remaining |M|−(|M−1|/2) keywords. The middle keyword, located at position |M|/2, is assigned to the parent node’s keyword. The parent node’s left pointer points to the left intermediate node, and the right pointer points to the right intermediate node. The current node pointer is set to the parent node, and the system returns to Step (2) to insert the next transaction until all transactions are inserted. For hash values stored in intermediate and root nodes, the system uses the Pointers, ChildNodeHashs, and TraceID to calculate the hash value. The system uses the blockchain version number, block index number, timestamp, difficulty calculation, the hash value of the previous block, and MB+ tree root to calculate the current block hash.

#### 5.1.2. External Index Creation

Each transaction inserted in the block must have a TraceID attribute. When we create the external index, the temporary traceability object table is first queried. If TraceID does not exist, then the TraceID is inserted into the temporary traceability object table. If the TraceID exists, we must update preTraceHash in the table pointing to the block with the newest traceability transaction.

To facilitate queries, we store the temporary traceability object table in a B+ tree. The insertion operation is similar to establishing an MB+ tree inside the block. Therefore, we do not further elaborate on the insertion algorithm’s operation.

### 5.2. Dual-Layer Index Query

The query process of the dual-layer index structure is divided into two parts, internal and external index query. The internal index queries the data in blocks, and the external index queries the temporary traceability object table. The overall traceability query process consists of two steps:

(1) We first query the temporary traceability object table according to the TraceID to find the latest transaction location. If the TraceID does not exist, an empty result is returned. Otherwise, the block with preTraceHash is searched.

(2) While searching the MB+ tree of the block, we try to find a leaf node with the same TraceID. Firstly, we determine whether the current node is a leaf node. If it is not a leaf node, the search is continued in the subtrees according to the relationship between the TraceID and the keyword of the current node. Then, if a leaf node is found, preTraceHash is evaluated. If preTraceHash is empty, the transaction in the leaf node is stored in the result set, which is returned to the client. Otherwise, Step (2) is executed according to preTraceHash.

## 6. Experiments

In this experiment, we compared the original non-layer index and the dual-layer index structure in terms of construction cost and traceability query efficiency. In this section, we introduce the software and hardware environment used during the experiment. Then, the experimental design and experimental results are presented.

### 6.1. Environment Setup

The native blockchain involves various complex components, such as consensus and cryptography. We want to clarify that these topics were beyond the scope of this study. Therefore, we built a simulation platform to conduct our experiment for the following reasons:

(1) The dual-layer index aims to alter the blockchain storage layer, not the entire blockchain framework. If the original blockchain framework was used, the cost of the whole system must be considered, including node consensus, block synchronization, etc. This would introduce more factors that could influence the analysis of experimental results.

(2) There is currently no available block storage module that solely focuses on blockchain storage, highlighting the need for this research.

### 6.2. Dataset Setup

The aim of the experiment was to evaluate the effectiveness of the dual-layer index structure and explore the factors that impact its construction time, storage, and query performance. Because these factors are related to the blockchain structure and not to the specific features of blockchain data, we generated the dataset used in the experiments. To simulate block data, the data format was designed to match that of the Bitcoin format, including a block header and body. The internal fields of the block header are consistent with Bitcoin’s, and the block’s internal structure is based on the MB+ tree. The specific attribute values of the transaction stored in the block did not affect the study purpose and were set as fixed-length random strings.

The amount of data is the most intuitive factor that affects query efficiency. We considered the number of traceability transactions, namely, the data amount, as the influencing factor and recorded it as $d_{i}$. The datasets used for our experiment are shown in Table 1.

Traceability queries consist of two steps, namely, data query within blocks and jumping between blocks. As such, the amount of traceability transaction data within blocks also affects the efficiency of the query. Therefore, the amount of traceability data within blocks, namely, the block size, is another influencing factor and is denoted as $b_{j}$. The block size is shown in Table 2.

The dataset is denoted as $d_{i}b_{j}$. Combined with different data amounts and block sizes, $d_{i}b_{j}$ has different block depths. The block depth refers to the total amount of blocks within the blockchain.

### 6.3. Experiment Setup

To assess the impact of the dual-layer index on both construction and query performance, we conducted four series of experiments. Each series consisted of two parts: evaluating the dual-layer index and comparing the dual-layer index with the non-layer index implementation, which was the native Bitcoin implementation.

(1) Block Construction Cost Experiment

We compared the block construction cost of the dual-layer index with the native Bitcoin block construction cost in two aspects: construction time and block storage usage. The experiment was divided into two groups. The first group consisted of $d_{i}b_{j}\;i\in \left\{1,2,3,4,5,6,7,8\right\}\;\mathrm{and}\;j\in \left\{3,5,7\right\}$ to verify the influence of the data amount on the block construction. The second group consisted of $d_{i}b_{j}\;i\in \left\{3,5,7\right\}\;\mathrm{and}\;j\in \left\{1,2,3,4,5,6,7,8\right\}$ to verify the impact of the block size on the block construction.

(2) Data Amount Influence Experiment

We aimed to evaluate the influence of the data amount on traceability query efficiency. In the beginning, we focused on the dual-layer index structure only. The datasets used were $d_{i}b_{j}$, where i∈{1,2,3,4,5,6,7,8} and j∈{3,5,7}. Then, we compared the traceability query efficiency between the dual-layer index structure and the non-layer index structure. The datasets used were $d_{i}b_{5}$, where i∈{1,2,3,4,5,6,7,8}. For all these cases, the query execution time was recorded for result analysis. In this experiment, the traceability query consisted of 1000 transactions. In each dataset, these data were randomly distributed.

(3) Block Depth Influence Experiment

According to the equation DataAmount=BlockSize×BlockDepth, we used the block depth to represent $d_{i}b_{j}$. We recorded the block depth as $p_{i}$. The datasets used for this experiment are shown in Table 3. Then, we recorded the number of transactions for the traceability querying, namely, the query case, as $q_{j}$. The datasets used for this experiment are shown in Table 4.

In this experiment, we aimed to analyze and compare the influence of block depth on query efficiency. To increase the depth of the block, we quantified the data to be queried and then added non-queried data to create a new dataset. Specifically, we utilized the dataset $p_{i}q_{j}$, where i∈{1,2,3,4,5,6,7,8} and j∈{1,2,3}, to evaluate and analyze the traceability query based on the block index method. Additionally, we selected $p_{i}$, where i∈{1,2,3,4,5,6,7,8}; and $q_{j}$, where j∈{2}, to verify the traceability query based on the dual-layer index and non-layer index methods.

(4) Block Size Influence Experiment

In this experiment, we compared and analyzed the effect of block size on query efficiency. Specifically, we utilized the dataset $d_{i}b_{j}$, where i∈{3,5,7} and j∈{1,2,3,4,5,6,7,8}, to evaluate and analyze the traceability query based on the block index method. Additionally, we utilized the dataset $d_{i}b_{j}$, where i∈{5} and j∈{1,2,3,4,5,6,7,8}, to verify the traceability query based on the dual-layer index and non-layer index methods. We then analyzed and compared the execution time of these methods. The distribution of the traceability data was similar to that in (2).

(5) Extreme Case Experiment

In this experiment, the aim was to evaluate the effectiveness of the dual-layer index structure with a large data amount. Thus, the large data amount was the influential factor, denoted as $D_{i}$. The set of large data used in this experiment is listed in Table 5.

(6) Complex Scenario Experiment

In real-world supply chain applications, different suppliers may provide raw materials with varying prices and qualities, which are mixed in batches to produce final products. During this process, there exist disassembly and assembly operations, which further complicate the traceability of the supply chain [38,39,40]. In the food supply chain, traceability entails the capacity to monitor and trace food, feed, food-producing animals, or substances intended for use in food or feed throughout all stages of production, processing, and distribution. As the supply chain involves several intermediaries between the initial grower of the food material and the end consumer, it is vital to document information on the movement of food items through intermediate processes.

During the intermediate processes, there can be a one-to-many or many-to-one relationship. This phenomenon is denoted as transaction bifurcation, where products are synthesized or decomposed. To address this issue at the application level, the corresponding upstream product source should be added to the product attribute. However, if the product attribute is restricted, a solution is sought at the index structure level. Namely, we need to modify the content of TraceID in the dual-layer index structure.

In our experiment, we investigated the one-to-many and many-to-one relationships between the upstream and downstream products. For the one-to-many relationship, we added extra suffix identifiers to the TraceID to differentiate between different downstream products. For many-to-one relationships, we included extra separators within the TraceID to distinguish between various upstream product sources. To account for the impact of transaction bifurcation, we denoted it as an influencing factor ($F_{i}$). The amount of bifurcation shows the number of upstream products involved in synthesis or the number of downstream products generated from decomposition. Table 6 presents the bifurcated dataset employed in our experiment. Additionally, we specified a fixed traceability length of 10, which could reflect the number of intermediaries involved in the bifurcation phenomenon.

### 6.4. Experimental Results and Analysis

This section provides details of our experimental results and analysis. In the experiments, we imported the generated dataset into two simulation systems: one based on the native blockchain architecture and the other based on the dual-layer index structure. In these simulation systems, blocks were stored as individual files on the disk, corresponding to a single file. The construction process involved writing the relevant datasets into the simulation systems and building the blocks accordingly. The traceability query process was performed by traversing all data or querying traceability objects based on the index. To clearly illustrate the experimental result, we introduced three indicator functions:

- C(L,M): block construction time on dataset L with index type M. While importing the dataset into the simulation system, we calculated the block generation time by obtaining the operation system’s timestamp.
- S(L,M): storage of the simulation system on dataset L with index type M. Once the data set was imported into the simulation system, we calculated the block storage occupancy by obtaining the storage size of the block files in the simulation system.
- T(L,M): execution time of traceability queries on dataset L with index type M. After importing the dataset into the simulation system, we conducted traceability queries on the simulation system and calculated the time required for traceability queries by obtaining the operation system’s timestamp.

In the above indicators, $M\in \left\{D,N\right\}$, in which D indicates the dual-layer index, and N indicates the non-layer index, namely the original blockchain structure. The dataset L refers to $d_{i}b_{j}$ or $p_{i}q_{j}$.

#### 6.4.1. Index Construction Cost Experiment

(1) Construction Time Result

**Figure 3:**
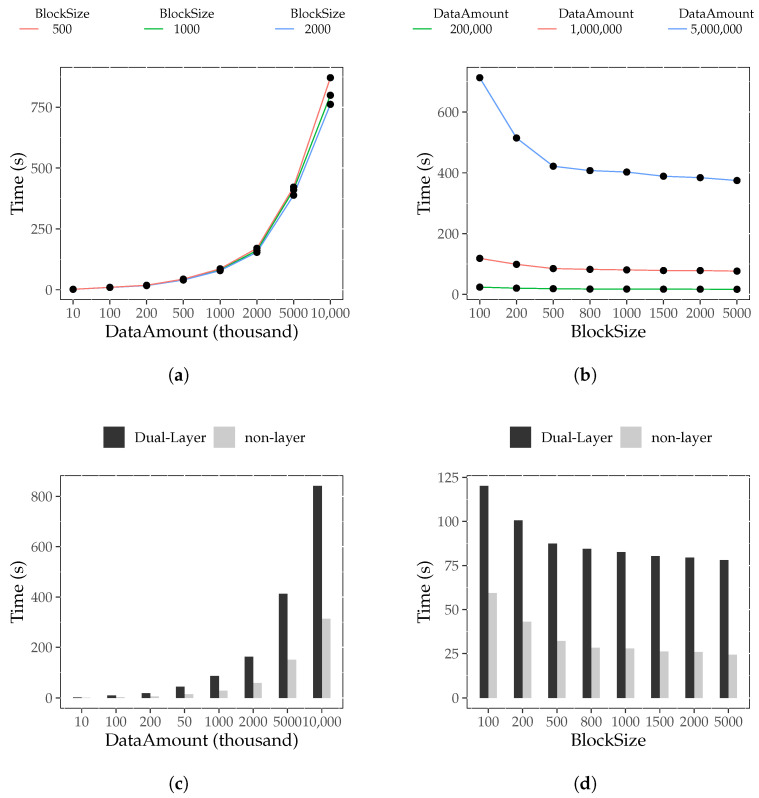
Experimental result regarding block construction time with dual-layer index under (**a**) different data amounts and (**b**) different block sizes; construction time comparison of the dual-layer index and non-layer index under (**c**) different data amounts and (**d**) different block sizes.

The results regarding the block construction time with the dual-layer index are presented in Figure 3a,b. It can be observed that $C(d_{i}b_{j},M)$ significantly increases with increasing *i* but gradually decreases with increasing *j*, with a diminishing rate of change. Therefore, we concluded that for a fixed block size, the data amount has a substantial impact on the block construction time. An increase in data amount leads to a longer block construction time. This is because the dual-layer index introduces time overhead, including maintaining temporary traceability tables and adding internal index fields. On the other hand, the block size has a weaker influence on construction time. When the data amount is constant, as block size increases, block construction time decreases. Because the data field of the block header is fixed and does not change with the block size, the larger the block size, the smaller the number of blocks under the same data amount, and the smaller the fixed block header. The smaller fixed block header reduces the time consumption, whereas a larger fixed block header increases the construction time consumption.

A comparison of the construction time between the dual-layer index and non-layer index is shown in Figure 3c,d. Normally, $C(d_{i}b_{j},D)$ is about 2.7 times larger than $C(d_{i}b_{j},M)$. Thus, we concluded that constructing a block with the dual-layer index takes approximately 2.7 times longer than that of a non-layer index block when the data size is kept constant. This is because the MB+ tree within the dual-layer index block needs to acquire data from the temporary traceability table and update it during the construction process.

(2) Block Storage Result

**Figure 4:**
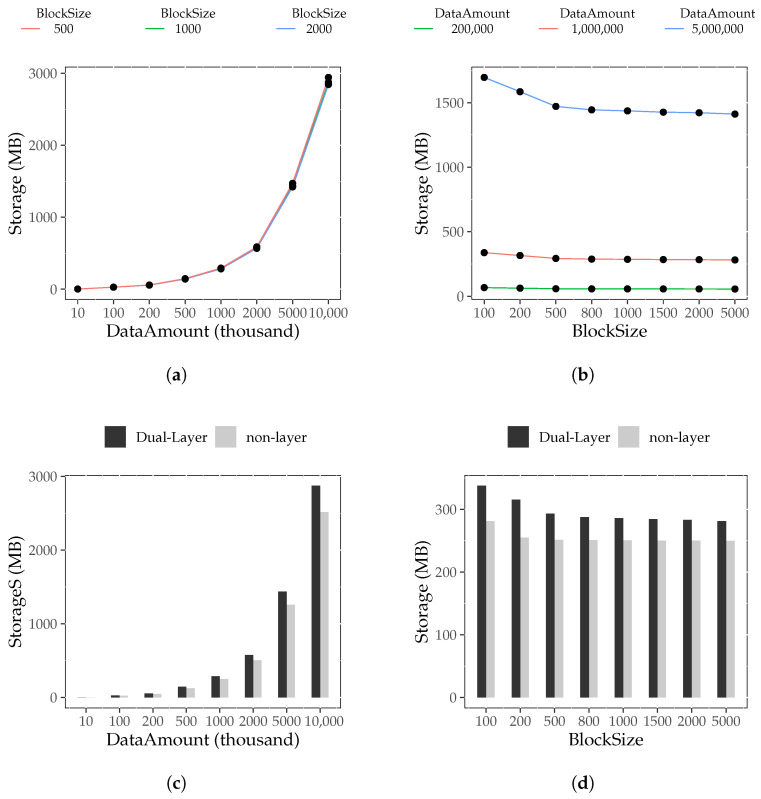
Experimental result of block storage usage of the dual-layer index under (**a**) different data amounts and (**b**) different block sizes; storage comparison of the dual-layer index and non-layer index under (**c**) different data amounts and (**d**) different block sizes.

The results for block storage usage are presented in Figure 4a,b. It can be observed that $S(d_{i}b_{j},M)$ significantly increases as *i* increases. On the other hand, $S(d_{i}b_{j},M)$ remains constant as *j* changes. Therefore, we concluded that, for a fixed block size, the data amount has a substantial impact on block storage use. This is because the dual-layer index introduces storage overhead, including the temporary traceability table and internal index fields. When the data amount is constant, the block size has little effect on the storage space needed after building the block. This is the influence of the fixed block header fields. The smaller the block, the larger the number of block headers, which increases the storage consumption. Otherwise, it reduces storage consumption.

The comparison of block storage usage between the dual-layer index and non-layer index is shown in Figure 4c,d. It can be observed that $S(d_{i}b_{j},D)$ is slightly larger than $S(d_{i}b_{j},N)$. Hence, we drew the conclusion that blocks with the dual-layer index take up slightly more storage space than blocks without the index for the same amount of data. The reason for this is that the root nodes and intermediate nodes of the MB+ tree in the dual-layer index block add pointer and keyword information based on the non-layer index tree. Additionally, the leaf nodes of the dual-layer index block add an external index and maintain a temporary traceability table, resulting in more storage space usage.

#### 6.4.2. Data Amount Influence Experiment

In this experiment, T-Dual-Layer ($d_{i}b_{j}$) was used for querying based on the block index and T-non-layer ($d_{i}b_{j}$) was used for query based on the non-layer index under the $d_{i}b_{j}$ test data set.

**Figure 5:**
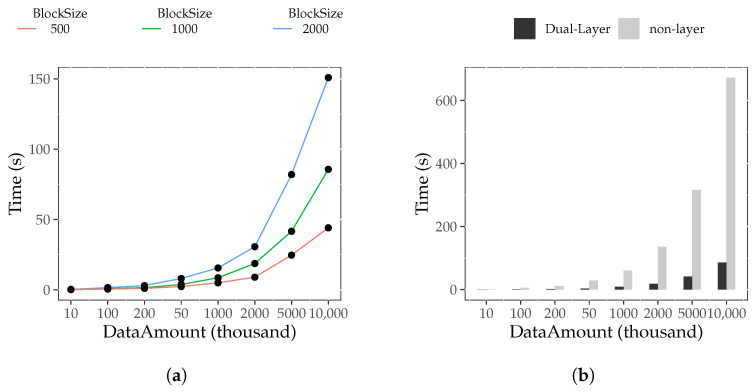
Experimental result of the influence of data size. (**a**) query time of dual-layer index under different data amounts; (**b**) query time comparison of dual-layer index and non-layer index under different data amounts.

Figure 5a shows the traceability query time of the dual-layer index ($T(d_{i}b_{j},D)$), where $i\in \left\{1,2,3,4,5,6,7,8\right\}$ and $j\in \left\{3,5,7\right\}$. It is observed that with the increase in *i*, $T(d_{i}b_{j},D)$ also increases. This indicates that for a fixed block size, the larger the amount of data, the more time required for a traceability query based on the dual-layer index. This is because the larger the data amount under a fixed block size, the larger the number of blocks. The query case is more likely to be distributed across multiple blocks, resulting in more time-consuming jumps through external indices.

Figure 5b shows a comparison of the traceability query time between the non-layer index ($T(d_{i}b_{j},N)$) and dual-layer index, where $i\in \left\{1,2,3,4,5,6,7,8\right\}$ and j=5. We can see that $T(d_{i}b_{j},D)$ is always smaller than ($T(d_{i}b_{j},N)$). When j=5, $T\left(d_{i}b_{j},D\right)\approx 7.5\times T\left(d_{i}b_{j},N\right)$. It shows that the efficiency of traceability queries based on the dual-layer index is much higher than that of the non-layer index.

#### 6.4.3. Block Depth Influence Experiment

**Figure 6:**
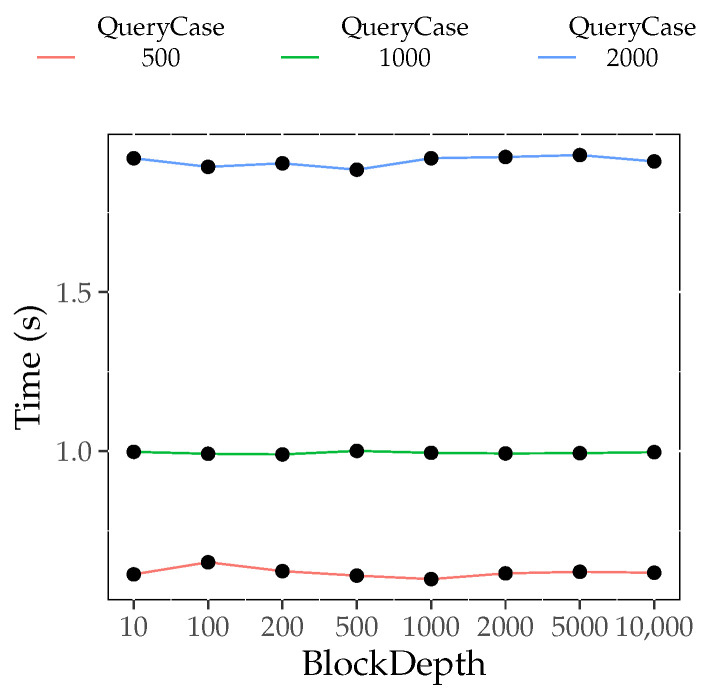
Query Time of the dual-layer index with different block depths.

Figure 6 shows the traceability query time of the dual-layer index ($T(p_{i}q_{j},D)$), where $i\in \left\{1,2,3,4,5,6,7,8\right\}$ and $j\in \left\{1,2,3\right\}$. It can be observed that with the increase in *i*, $T(p_{i}q_{j},D)$ is unchanged under different *j* values. This indicates that, for a fixed query case, the traceability query time is independent of the block depth based on the dual-layer index. This is because the external index records the latest trace object. No matter how much data are appended, the index query directly starts from the last place where the trace object appears. It can also be observed that $T(p_{i}q_{j},D)$ increases with increasing *j*. This indicates that the more data in the query case, the more time the traceability query takes. This is because, at the same block depth, more data in the query case lead to a longer query process.

Table 7 compares the traceability query execution time between the non-layer index and dual-layer index for various block depths. From the table, it is evident that, except for the first case (BlockDepth = 10), the execution time for the dual-layer index is consistently lower than that for the non-layer index for the same block depth. This is because as the block depth increases, the blockchain becomes longer. However, the dual-layer index avoids a one-by-one block query through the block jump process. Thus, increasing the length of the blockchain does not affect the traceability query time of the dual-layer index for specific target data queries. On the other hand, because queries on the non-layer index require a block-by-block comparison, the traceability query efficiency decreases as the length of the blockchain increases.

#### 6.4.4. Block Size Influence Experiment

**Figure 7:**
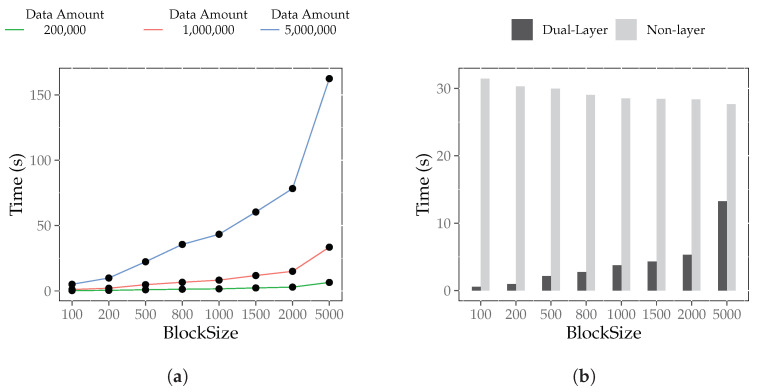
Experimental result of the influence of block size. (**a**) Query time of dual-layer index for different block sizes; (**b**) Query time comparison of the dual-layer index and non-layer index for different block sizes.

Figure 7a illustrates the effect of block size on the traceability query time of the dual-layer index. As the results show, as *j* increases, $T(d_{i}b_{j},D)$ also proportionally increases, indicating that larger block sizes result in longer traceability query times for the dual-layer index. This is because larger block sizes increase the number of jumps between blocks, which slows the query.

Figure 7b compares the traceability query time between the non-layer index and the dual-layer index for different block size configurations. Notably, the traceability query time $T(d_{i}b_{j},D)$ is consistently lower than $T(d_{i}b_{j},N)$. Moreover, when *i* is kept constant, $T(d_{i}b_{j},N)$ decreases as *j* increases while $T(d_{i}b_{j},D)$ increases. This observation suggests that the traceability query efficiency of the non-layer index improves as block size increases. The traceability query efficiency of the dual-layer index decreases for larger block sizes for a given amount of data. This is because the dual-layer index structure primarily optimizes the block jump process. As the data amount increases, the number of blocks decreases, and the time required for block jumps in the traceability query process of the non-layer index structure decreases, thereby reducing the optimization effect of the dual-layer index on traceability queries.

#### 6.4.5. Extreme Case Experiment

Table 8 presents the query time, construction time, and storage occupation of the dual-layer index structure for various large datasets. Our experimental findings suggest that even with large data amounts, the query time, construction time, and storage occupation of the dual-layer index structure exhibit linear variation. This indicates that the dual-layer index structure can efficiently process large data amounts, as demonstrated by our experimental results.

#### 6.4.6. Complex Scenario Experiment

Table 9 presents the execution time of traceability query under complex scenarios such as the one-to-many scenario and the many-to-one scenario.

In the one-to-many scenario, the query time remains constant for different bifurcation factors. This finding reveals that the traceability time in a one-to-many scenario is independent of the bifurcation factor when the traceability length is constant. This can be attributed to the fact that in a one-to-many relationship, the initial product can be perceived as gradually decomposing into subproducts. Despite the number of subproducts generated, the traceability from any subproduct always returns to the same length and, ultimately, back to the source product, thereby keeping the query time unchanged.

In the many-to-one scenario, the query time increases with the increase in the bifurcation factor. This finding indicates that the traceability time in the many-to-one scenario positively correlates with the bifurcation factor when the traceability length is constant. In a many-to-one relationship, the product is perceived as gradually merging with multiple raw materials. The presence of multiple raw materials requires searching for upstream raw materials based on the final merged product, which necessitates multiple backtracking. The larger the number of bifurcation factors, the more raw materials are involved, and the more backtracking is required, leading to an increase in the query time.

In practical production activities, it is common to find one-to-many and many-to-one relationships in supply chains. However, mixed queries are local traceability queries involving one-to-many and many-to-one relationships. Thus, the fundamental principles remain unchanged.

## 7. Discussion

This section starts with a summary of our experimental results. Then, we illustrate the implementation scenarios of the dual-layer index structure in a practical environment. Furthermore, we compare our work with other blockchain-based supply chain traceability research. In the end, we discuss the assumptions and limitations of our method.

### 7.1. Discussion of Experimental Results

The construction of a dual-layer index structure incurs additional time and space overhead. Based on the experimental results presented in Section 6.4.1, we concluded that the 2.5 to 3.4 times more time overhead is required for constructing a dual-layer index structure compared with the original blockchain structure. However, the gap in time overhead narrows as the block size increases. Similarly, the space overhead is 1.1 to 1.2 times greater than that of the original blockchain structure, and the gap decreases as the block size increases. Though there is overhead, constructing a dual-layer index structure can significantly enhance the efficiency of traceability queries. As seen from the experimental results in Section 6.4.2 and Section 6.4.4, the traceability query efficiency under the dual-layer index structure is 7.1 to 7.9 times higher than that of the original blockchain when the traceability data account for a certain proportion (set at 10% in this experiment) of the total data. The gap in query efficiency increases as the block size decreases. In contrast, the experimental results in Section 6.4.3 show that the query efficiency under the dual-layer index structure is determined by the amount and distribution of traceability data rather than the total amount of data. The experimental results in Section 6.4.5 indicate that the dual-layer index structure can efficiently process large amounts of data, as demonstrated by the linear variation observed in the query time and storage occupation for varying dataset sizes. Section 6.4.6 shows the query time is independent of the bifurcation factor in the one-to-many scenario. In contrast, the query time increases with the increase in the bifurcation factor in the many-to-one scenario. In a one-to-many scenario, the traceability from any subproduct always returns to the same length and ultimately back to the source product; in a many-to-one scenario, the presence of multiple raw materials requires multiple backtracking, which can be interpreted as multiple traceability queries.

In general, the dual-layer index structure changes the process of obtaining traceability data by replacing block traversal in the original blockchain with external block jumps based on the external index and fast queries within blocks based on the internal index. The dual-layer index reduces query time and improves query efficiency for traceability data. Although constructing the index incurs some time overhead and requires slightly more storage space, the benefits of improving query efficiency for traceability data far exceed these costs.

### 7.2. Implementation Discussion

The internal index requires modifications of the block structure of blockchains, which makes it impractical to directly implement the dual-layer index in practical applications. Two scenarios for the implementation of the dual-layer index structure are proposed.

- For a novel food supply chain, the initial step is to modify the MB+ tree block structure. This alteration is imperative for storing the internal index and a part of the external index. Following this, the location for storing the external index must be determined. Three main options exist: (1) If a blockchain system supports a state database, such as Fabric, Ethereum, or Partiy, the external index can be stored within this database. (2) Another option is to make use of a special block that is designated for external index storage, but this may lead to query time overhead. (3) Alternatively, an external database can be introduced to store the external index, though this approach may introduce security and tampering problems. In the end, to ensure the maintenance of the system, an intelligent contract must be created that writes the traceability data and effectively maintains the dual-layer index structure.
- For an existing food supply chain, the first step is to create a novel blockchain environment that supports the dual-layer index feature. Subsequently, the traceability data should be exported from the original supply chain system and imported into the novel blockchain system. However, this process may entail a cross-chain operation. If the underlying blockchain systems are identical, a data transformation intelligent contract would be necessary. Otherwise, the traceability data have to be exported to local storage and imported to the novel blockchain system later. However, the use of local storage for data exporting poses potential issues. The absence of adequate data protection mechanisms may result in tampering problems.

To illustrate the implementation details, we introduced a case study based on the European food supply chain, a blockchain system for ensuring traceability, as described in [41]. The system allows consumers to reconstruct the entire history of a product up to its origin to verify its health and quality through a simple QR code scan. The general agricultural food supply chain consists of all activities at different stages, namely, production, processing, distribution, and consumption. The proposed solution considers the continuity of different stages and events in the supply chain. Following the four stages mentioned above, the case study was a rice supply chain.

(1) In the production stage, farmers add crop information to the blockchain, including the seed source, production site, fertilizer use, harvest time, product batch, etc. At this stage, a TraceID is defined, which can be defined based on the production site and production batch. Rice produced in the same location and batch is assigned the same TraceID.

(2) In the processing stage, processors first store transaction records with farmers on the blockchain, including information on both the parties and the traded items. Then, processors need to store information about the rice in the drying, cleaning, and storage processes, including the product batch, use of chemicals, impurity content, removal rate, inventory number, product source, storage time, quality inspection report, product category, product quantity, etc. In the processing stage, rice may undergo branching or merging due to different storage and processing batches. For example, rice with different TraceIDs may be stored under the same conditions, or rice with the same TraceID may undergo different processing batches. In this case, the TraceID of the branching product can be identified by adding an additional identifier. The TraceID of the merged product can be separated by a delimiter, indicating that the merged result comes from rice with multiple TraceIDs.

(3) In the distribution stage, distributors first store transaction records with processors on the blockchain, including information on both parties and the traded items. Distributors then record transportation details, tracking information, storage conditions (such as temperature and humidity), and transportation time. All transactions between distributors and retailers, as well as all relevant information, are written into the blockchain platform. In this stage, packaged rice may also undergo branching or merging due to the different transportation processes. The handling of the TraceID at this stage is the same as in the processing stage.

(4) In the consumption stage, detailed information about each bag of rice is recorded, including its current quality, expiration date, and storage conditions. The TraceID from the distribution process is used in this stage and is recorded on the bar code attached to the final product packaging. Customers who purchase the rice can scan the bar code to obtain the TraceID and trace the origin of the rice.

To analyze this case using a Hyperledger-Fabric-based dual-layer indexing structure, we needed to make technical modifications to the Hyperledger Fabric blockchain. Based on the characteristics of Hyperledger Fabric, the main modifications can be divided into three parts: modifications of the block structure, modifications of interface implementations, and additional interface implementations.

(1) Modifications of the block structure: The block structure in Hyperledger Fabric consists of a block header and a block body. The block header does not require any modifications, and the dual-layer indexing structure only needs to modify the storage structure of the transaction data in the block body to an MB+ tree structure. External indices are introduced into the stored transaction data in the block body through two new fields: TraceID and preTraceHash. Because the Hyperledger Fabric chaincode Query System Chaincode (QSCC) comes with the GetBlockByHash interface, block data can be directly retrieved by the block hash. Therefore, the preTraceHash can assist block-to-block jumping, reducing the modification cost. (a) Users provide the TraceID that is recorded in the QR code or bar code of the product. The TraceID is stored in the system as product information is recorded. For example, in the rice supply chain, farmers in the production stage, processors in the processing stage, distributors in the distribution stage, and retailers in the consumption stage need to provide the TraceID when recording intermediate product information on the blockchain. (b) preTraceHash is updated with the temporary traceability table. Hyperledger Fabric supports a state database, so the table in the dual-layer index structure is stored in the state database. The table contains information such as TraceID, TransactionID, and preTraceHash. When a transaction with a corresponding TraceID is first inserted, the preTraceHash is null. During the Fabric transaction process, the orderer nodes generate block sorting, and the peer node synchronizes the block data to the state database. Therefore, the source code of the synchronization process needed to be modified to synchronize TraceID, TransactionID, and preTraceHash to the state database during this process.

(2) Modifications of interfaces: Modifications of the block structure affect the interfaces related to block operations. In Hyperledger Fabric, some interfaces are based on the state database to obtain data, such as PutState and GetState. Because the storage format of the state database remains unchanged, these interfaces do not need to be modified. However, some interfaces are based on block queries in the QSCC, such as GetBlockByNumber, GetTransactionById, GetBlockByHash, etc. Due to the modifications in the block structure, the interface implementation of QSCC needed to be modified.

(3) Additional interface implementations: To improve the efficiency of traceability queries of the dual-layer index structure, an additional traceability query interface needed to be implemented. The traceability query interface was designed to query the complete history of a product based on its TraceID. The query process involves two parts: (a) The first part involves finding the block with the last TraceID based on the temporary traceability table in the state database, parsing the block data, and searching transactions with the same TraceID using the MB+ tree query. (b) The second part involves jumping to the corresponding block for the query based on the preTraceHash. This step is repeated until the preTraceHash field of the transactions in the result set is null. To integrate these two steps into a query interface, the first step can be implemented using the Fabric native interface GetState, as the temporary traceability table is stored in the state database. The second step can be implemented using the GetBlockByHash interface in QSCC, which retrieves the corresponding block data and parses the transaction set. By implementing these two steps in a query interface, users can easily query the complete history of a product based on its TraceID.

Combining the Fabric transformation based on the dual-layer index structure, we calculated the transformation cost. Regarding the cost of modifying the code, the dual-layer index structure involves modifying the block body structure; it is necessary to insert the TraceID, TransactionID, and preTraceHash fields of the temporary traceability table into the synchronous block data. However, in Fabric, changing the block body structure affects the code modification of the block, including block data storage and query. Therefore, the following source code needs to be modified: the block body structure, block storage format, block data synchronization, etc. The modification involves the following packages: common package, core package, and peer package, resulting in a large range of modifications. Fabric is a mature blockchain technology with a complete identity authentication mechanism, encryption mechanism, and consensus algorithm. These mechanisms are unrelated to the block structure, and the dual-layer index structure does not involve modifying the above mechanisms. Therefore, the code modification introduced by the dual-layer index structure accounts for a relatively small amount of Fabric source code.

Regarding the maintenance cost, after the block structure transformation, the data stored in the block follow the standard transaction insertion logic without additional user operation. However, the temporary traceability table must be maintained in real-time. Specifically, the TraceID of the temporary traceability table needs to be updated whenever the transaction is inserted. Regarding storage cost, in our study case, the native Fabric occupies 36.4 GB, while the Fabric with the dual-layer index occupies 45.9 GB, approximately 1.26 times larger the original block storage cost. This result is consistent with the general experimental results. Regarding query performance improvement, the original Fabric stores case data and performs traceability queries. It takes 1534.16 s to analyze data by traversing blocks, whereas it takes only 375.53 s to use the additional traceability query interface for traceability queries under the dual-layer index structure. However, in actual cases, traceability queries require network communication between clients and the blockchain, which consumes extra time due to network I/O compared with the experimental results.

### 7.3. Comparison with Related Methods

We compared the dual-layer index structure with related methods based on method type, security, sensitive data protection, storage overhead, and traceability query influencing factors. The comparison results are summarized in Table 10.

In addition to our method, those of Lai et al. [42] and Qin et al. [43] use an index structure to optimize traceability query efficiency. Lai et al. [42] designed an index structure independent of the Merkle tree to support efficient intrablock queries and create new fields in the block header to optimize the interblock query. However, the method of Lai et al. still needs to traverse all the blocks to compare the added block header fields. The method of Qin et al. [43] also needs to find the first traceability object by traversing the block. Moreover, their experiments showed that the efficiency of the traceability query is related to the total amount of data under their indexing methods. The advantage of our method is that traceability time is only affected by traceability length.

Li et al. [26] and Peng et al. [14] achieved the advancement of additional query middleware in the blockchain, but both studies introduced external database storage, which may cause security problems.

Martintoni et al. [44] proposed a sensitive data protection strategy based on an authentication mechanism in an alliance chain application. Meanwhile, a more comprehensive sensitive data management approach was developed by Cha et al. [45], which adopts a key escrow encryption system for data protection. Notably, the dual-layer index structure only makes modifications to the blockchain structure to boost the performance of the traceability query, thereby preserving the inherent features of the blockchain. Therefore, we did not consider the sensitive data projection in the dual-layer index structure. The aforementioned data protection strategies are complementary to our method.

### 7.4. Assumptions and Limitations

After analyzing the characteristics of the dual-layer index structure and reviewing the experimental results, we identified several assumptions and limitations of this approach.

(1) Assumptions

Firstly, the implementation of the dual-layer index structure requires a transformation of the blockchain to accommodate changes in the block structure. This transformation can be directly upgraded for private and alliance chains; however, for public chains, the requisite technical transformation authority must be obtained.

Secondly, it is recommended to have technical support similar to the state database, which is readily available in many existing blockchain technologies such as Fabric and Ethereum. By using a state database, the external index can be stored in it for maintenance, maximizing the benefits of the dual-layer index structure. However, if a stateless database is used, there might be a need to store the external index in the block, which could result in a performance decrease.

Thirdly, the benefits of the dual-layer index structure are particularly evident when there is a significant user base. It is easy to distribute traceability data across nonadjacent blocks and enhance the efficiency of queries.

Fourthly, it is essential to determine an appropriate block size. When the total data amount is fixed, a smaller block size leads to a larger number of blocks. Because each block contains an MB+ tree structure (internal index), this results in increased overhead storage. Conversely, a larger block size leads to a higher MB+ tree and reduces the number of blocks needed. However, the effectiveness of the dual-layer index structure is weakened due to longer intrablock searches and fewer block jumps during traceability queries. Therefore, it is necessary to make a trade-off decision.

(2) Limitations

The security and tampering issues of the dual-layer index structure are dependent on the security features of the underlying blockchain technology. The internal index MB+ tree retains the properties of the Merkle tree, allowing it to expose data tampering in a timely manner. Likewise, the temporary traceability tables rely on the storage of the blockchain itself. Therefore, the dual-layer index structure does not introduce any additional security risks to the blockchain, and its security is entirely dependent on the reliability of the underlying blockchain technology.

One potential issue with the dual-layer index structure is the lack of protection mechanisms for data privacy, especially sensitive data. The data involved in the supply chain include not only product traceability information but also private data that only related enterprises can access, such as transaction information. For competitive enterprises, data privacy is a crucial issue. The external index uses the self-increasing TraceID as the index’s primary key. This may lead to the leakage of sensitive data, as tracing all products’ specific traceability paths can be easily achieved at a low cost (by traversing the external index), which could expose the cooperative relationships between companies. One solution to this issue is to introduce other TraceID-encoding schemes or encryption methods to encrypt the TraceID. The TraceID could then be accessed through specific decoding means when tracing the product’s traceability path, which can effectively protect sensitive data and ensure that only authorized parties can access traceability information.

Under extreme traceability scenarios, the storage cost can sharply increase. When the total amount of data is fixed, an increase in traceable data leads to an increase in external index storage costs. Additionally, when there are too many traceability branches, the TraceID in the external index may contain a large number of repeated prefixes, exacerbating external index storage costs. In the case of one-to-many traceability, a snowflake-like pattern may emerge, further increasing storage costs. For many-to-one traceability, using separators to identify upstream products can make the internal index invalid. To address these challenges, a new external TraceID index coding method could be introduced. At the application level, the TraceID of upstream products can be recorded as part of product attributes. This can help to reduce storage costs and ensure the external index remains accurate and efficient, even in extreme traceability scenarios.

## 8. Conclusions

In recent years, food quality and security issues have become increasingly serious. Ensuring transparency and traceability in the food supply chain is crucial for maintaining food safety, quality control, compliance, and consumer trust. Compared with traditional centralized management approaches, blockchain technology offers a better solution with its decentralized and traceable characteristics.

To this end, we proposed a dual-layer index based on blockchain that can be applied to supply chain traceability systems, providing an efficient solution to support traceability. We designed an MB+ tree data structure to improve the internal block index and used a temporary traceability table as the external block index. Our experiments demonstrated that the traceability query capability of the dual-layer index is significantly improved, with an average increase in traceability query efficiency of seven to eight times. Furthermore, we verified that the dual-layer index structure can function generally under large datasets and complex scenarios. The results of these experiments demonstrated that the query efficiency of the dual-layer index structure in complex scenarios still adheres to general patterns and is only related to traceability length.

To effectively implement the dual-layer index structure for supply chain traceability, several assumptions must be met, including transformation conditions corresponding to blockchain technology, support for the state database, a sufficient number of users, and reasonable block size parameters. Meeting these assumptions is essential to maximize the benefits of the dual-layer index structure while minimizing overhead costs and ensuring efficient storage and retrieval of traceability data. The dual-layer index structure also has some limitations, which need to be addressed. The security of the structure entirely depends on the underlying blockchain technology, and sensitive data may be exposed if not protected. The temporary traceability table may reveal the cooperative relationships between companies, but this can be addressed by encoding or encrypting the TraceID. In extreme traceability scenarios, the structure may introduce significant storage overhead, requiring storage data or modifying the TraceID-encoding method at the application level.

In future work, we aim to improve the dual-layer index structure by addressing its existing limitations. These improvements include enhancing the protection of sensitive data by incorporating existing sensitive-data-protection methods, developing a new TraceID encoding method combined with cryptography techniques, and investigating new storage structures to improve its scalability while maintaining query efficiency. These enhancements aim to enhance the security, scalability, and efficiency of the dual-layer index structure, making it more applicable for practical use. Furthermore, we plan to explore integrating index structures with product attributes in the future, which may involve incorporating new and emerging data formats. Such integration could help minimize the storage overhead of additional indices currently in use. 

## Figures and Tables

**Table 1:** Configuration of data amount.

$d_{1}$	$d_{2}$	$d_{3}$	$d_{4}$	$d_{5}$	$d_{6}$	$d_{7}$	$d_{8}$
$10^{4}$	$10^{5}$	$2\times 10^{5}$	$5\times 10^{5}$	$10^{6}$	$2\times 10^{6}$	$5\times 10^{6}$	$10^{7}$

**Table 2:** Configuration of block size.

$b_{1}$	$b_{2}$	$b_{3}$	$b_{4}$	$b_{5}$	$b_{6}$	$b_{7}$	$b_{8}$
$10^{2}$	$2\times 10^{2}$	$5\times 10^{2}$	$8\times 10^{2}$	$10^{3}$	$1.5\times 10^{3}$	$2\times 10^{3}$	$5\times 10^{3}$

**Table 3:** Configuration of block depth.

$p_{1}$	$p_{2}$	$p_{3}$	$p_{4}$	$p_{5}$	$p_{6}$	$p_{7}$	$p_{8}$
$10^{1}$	$1\times 10^{2}$	$2\times 10^{2}$	$5\times 10^{2}$	$10^{3}$	$2\times 10^{3}$	$5\times 10^{3}$	$5\times 10^{4}$

**Table 4:** Configuration of query case.

$q_{1}$	$q_{2}$	$q_{3}$
$5\times 10^{2}$	$1\times 10^{3}$	$2\times 10^{3}$

**Table 5:** Configuration of large amounts of data.

$D_{1}$	$D_{2}$	$D_{3}$	$D_{4}$
$2\times 10^{7}$	$5\times 10^{7}$	$10^{8}$	$2\times 10^{8}$

**Table 6:** Configuration of bifurcation amount.

$F_{1}$	$F_{2}$	$F_{3}$	$F_{3}$
1	2	3	4

**Table 7:** Query time comparison of dual-layer index and non-layer index for different block depths.

Block Depth	10	100	200	500	1000	2000	5000	10,000
non-layer Index (s)	0.686	6.04	11.975	30.003	58.634	115.321	284.665	658.633
Dual-Layer Index (s)	0.998	0.992	0.99	1.001	0.995	0.993	0.994	0.997

**Table 8:** Query time, construction time, and construct storage of dual-layer index with different data amounts.

Data Amount	$2\times 10^{7}$	$5\times 10^{7}$	$10^{8}$	$2\times 10^{8}$
Query Time (s)	165.816	423.128	816.563	1655.432
Construction Time (s)	1579.213	3910.875	7936.125	17,136.263
Construct Storage (GB)	6.58	14.13	29.16	61.59

**Table 9:** Query time comparison of one-to-many and many-to-one for different bifurcation factors.

Bifurcation Factor	1	2	3	4	5
One-To-Many	0.124	0.118	0.102	0.136	0.122
Many-To-One	0.135	1.545	42.598	469.312	3289.885

**Table 10:** Comparison results.

Ref	Research Object	Method Type	Security	Sensitive Data Protection	Storage Overhead	Traceability Query Influencing Factors
Ours	Blockchain Traceability Query	Index	Blockchain	No	Low	Traceability Length
[42]	Blockchain Traceability Query	Index	Blockchain	No	Low	Data Amount
[43]	Blockchain Traceability Query	Index	Blockchain	No	Low	Data Amount
[26]	Blockchain Query	External Database	External Database	No	High	Data Amount
[14]	Blockchain Query	External Database	External Database	No	High	Data Amount
[44]	Application of Supply Chain	Application	Blockchain	Yes	Low	-
[45]	Application of Supply Chain	Application	Blockchain	Yes	Low	-

## Data Availability

Data are contained within the article.
